# Evolution of Bacterial Tolerance Under Antibiotic Treatment and Its Implications on the Development of Resistance

**DOI:** 10.3389/fmicb.2021.617412

**Published:** 2021-02-26

**Authors:** Jordy Evan Sulaiman, Henry Lam

**Affiliations:** Department of Chemical and Biological Engineering, The Hong Kong University of Science and Technology, Kowloon, Hong Kong

**Keywords:** tolerance, resistance, persistence, laboratory evolution, antibiotic

## Abstract

Recent laboratory evolution studies have shown that upon repetitive antibiotic treatments, bacterial populations will adapt and eventually became tolerant and resistant to the drug. Drug tolerance rapidly evolves upon frequent, intermittent antibiotic treatments, and such emerging drug tolerance seems to be specific to the treatment conditions, complicating clinical practice. Moreover, it has been shown that tolerance often promotes the development of resistance, which further reinforces the need of clinical diagnostics for antibiotic tolerance to reduce the occurrence of acquired resistance. Here, we discuss the laboratory evolution studies that were performed to track the development of tolerance in bacterial populations, and highlight the urgency of developing a comprehensive knowledge base of various tolerance phenotypes and their detection in clinics. Finally, we propose future directions for basic research in this growing field.

## Distinction Between Bacterial Tolerance and Resistance as Survival Mechanisms Toward Antibiotic Treatment

Bacterial cells have a multitude of ways to fight against antibiotic assault. The most well-studied one is resistance (D’Costa et al., 2006), in which the bacteria possess genetic mutations that protect themselves against certain types of antibiotics, thus allowing them to grow at higher antibiotic concentrations. Mechanisms for resistance include direct inactivation of the drug, alterations of drug targets to reduce binding affinity, decreasing uptake or increasing efflux, redundant pathways to bypass the affected drug targets, and many more (Giedraitienė et al., 2011; Cox and Wright, 2013). In general, resistance directly counters the antibiotic’s action mechanism. The most common detection method for resistance is through the measurement of the minimum inhibitory concentration (MIC), which is the lowest concentration that would kill or inhibit the growth of bacteria (Wiegand et al., 2008). A resistant population would have an elevated MIC. If the resistance phenotype only occurs in a subpopulation of cells, then it is known as heteroresistance. Heteroresistance is often unstable (Andersson et al., 2019): when grown in the absence of antibiotics within a limited number of generations, the phenotype reverts to susceptibility, while in the presence of antibiotic, the resistant subpopulation would rapidly outcompete the sensitive cells, causing an ambiguous classification of the population as resistant. Unstable heteroresistance can be caused by an intrinsic instability of the resistance mutation itself, or by genetically stable mutations that confer high fitness cost. In the former case, the resistant subpopulation contains an increased copy number or tandem amplification of genes that increase resistance. In the latter case, resistance mutations with high fitness cost often drive the selection of second-site compensatory mutations when grown in the absence of antibiotic pressure, which will reduce the cost, but also lead to the loss of resistance. Although the majority of the population will become susceptible (harboring both resistance mutation and compensatory mutation which increase fitness), there will still be a small fraction of resistant cells (with reduced fitness, containing only the resistance mutation).

Another way for bacteria to survive antibiotic assault is tolerance. A tolerant population exhibits no difference in the MIC compared to a susceptible population, but can survive high doses of bactericidal antibiotics, often much higher than the MIC (Brauner et al., 2016; Balaban et al., 2019). Unlike resistance that allows the cells to grow under higher antibiotic concentrations, tolerant populations cannot grow nor replicate during treatment, and are just being killed at a lower rate. Tolerance can be quantified by measuring the minimum duration of killing (MDK) of the population, namely the time it takes to reduce the population by a certain percentage (e.g., 99%) at a certain dose of antibiotic (Fridman et al., 2014). If the tolerance phenotype only occurs in a subpopulation of cells, then it is known as persistence or heterotolerance (Lewis, 2010; Brauner et al., 2016). The tolerant subpopulation, called persisters, are present naturally in almost every bacterial populations. Persistence is known to be a phenotypic state rather than a genetic trait (Sulaiman et al., 2018), and is often interpreted as a bet-hedging strategy of bacteria to position some “seed” cells in a population to survive and outlive unfavorable environmental conditions (Lennon and Jones, 2011). Several mechanisms and pathways have been implicated in the phenotypic switch to a persister state, such as the stringent response and (p)ppGpp signaling, RpoS and the general stress response, SOS response, bacterial communication and quorum sensing, and Toxin/Antitoxin (TA) modules (Harms et al., 2016; Michiels et al., 2016b). More recently, it was proposed that the mechanistic basis of persister formation and resuscitation is via ppGpp ribosome dimerization (Song and Wood, 2020; Wood and Song, 2020). In short, ppGpp directly generate persister cells by inactivating ribosomes through stimulation of the ribosome modulation factor (Rmf), hibernation promoting factor (Hpf), and ribosome-associated inhibitor (RaiA). Upon addition of nutrients and removal of stress, cAMP levels are reduced and HflX is produced, causing the dissociation of inactive ribosomes into active ribosomes which leads to the resumption of growth. However, the exact mechanism of how persisters survive high-dose antibiotic treatment remains an open question and is likely antibiotic-dependent, though it is surmised that it can be partially ascribed to dormancy, which renders the killing mechanisms of many antibiotics ineffective (Wood et al., 2013; Kotte et al., 2014). Although persister cells do not differ genetically from their susceptible counterparts in the same population, it has been shown that the “level of persistence” – namely, the propensity of a strain to form persisters – can be modulated by genetic changes (Moyed and Bertrand, 1983; Germain et al., 2013). The presence of persisters explains the biphasic killing pattern when bacteria are treated with bactericidal antibiotics. The first phase with the steeper slope marks the rapid decline of the susceptible cells, and the second phase indicates the slow decline of the persister cells (Sulaiman and Lam, 2019).

## Laboratory Evolution to Study the Development of Tolerance in Bacteria

Bacteria are well-known for their ability to adapt to different environmental conditions. When subjected to transient stresses, subpopulations of cells with favorable phenotypic niches that are otherwise outcompeted under normal conditions (such as those that have a slower growth), may thrive. This subpopulation may possess genetic mutations that confer tolerance or a higher level of persister formation, but with a certain fitness cost associated with the higher fraction of persister cells. When similar stresses are applied repeatedly, these cells could be selected, leading to an increase in the level of tolerance of the population over time. Therefore, the resulting evolved population may have different physiology and behavior compared to the original one. Recently, there have been substantial efforts devoted to study this adaptation mechanism and the development of tolerance in bacteria through laboratory evolution experiments, where bacterial populations are repetitively treated with high doses of antibiotics, mimicking clinical conditions (Fridman et al., 2014; Mechler et al., 2015; Michiels et al., 2016a; Van den Bergh et al., 2016; Khare and Tavazoie, 2020; Sulaiman and Lam, 2020a). One of the first experiments that inspired the laboratory evolution strategy came from Moyed et al. They repetitively treated *E. coli* cells with ampicillin and identified the *hipA* gene that confers a high persistence phenotype (Moyed and Bertrand, 1983). The increased tolerance upon laboratory evolution experiment was not only observed in *E. coli*, but also in *Staphylococcus aureus* (Mechler et al., 2015) and other ESKAPE pathogens (Michiels et al., 2016a), indicating the seemingly universal adaptability of bacteria toward intermittent antibiotic treatments. These laboratory evolution experiments, combined with theoretical models (Kussell et al., 2005; Patra and Klumpp, 2013), suggested that such cyclic antibiotic treatment protocols, commonly practiced in clinics, should allow for tolerant cells to eventually take over the population.

Tolerance mutations may be caused by the antibiotic treatment itself, or arise spontaneously. Either way, the small number of tolerant mutants would stay hidden and undetected in the population under normal growth conditions, but survives better when the antibiotic is present and hence would be able to take over the population during the course of repetitive antibiotic treatments. This effect could be predicted by the mathematical model of persistence described by Balaban et al. (2004) and Gefen and Balaban (2009), if one extended it to include a high-persistence mutant, whereby the mutant possesses a higher conversion rate to persisters, but is otherwise identical to the wild-type (Supplementary Figure 1a). This mutant, by itself, would have a tolerant phenotype due to a higher fraction of persisters. If this mutant is mixed with a wild-type strain and subjected to intermittent antibiotic treatments, the resulting population dynamics can be simulated in an evolutionary model, with alternating periods of killing and regrowth (Van den Bergh et al., 2016; Sulaiman and Lam, 2020a; Supplementary Figure 1b). In this scenario, the simulation showed that the mixed population would indeed attain a higher and higher survival rate against the antibiotic after several cycles, which can be traced to the invasion of the small subpopulation of the tolerant mutant. Namely, since the mutant has a higher propensity to convert to persister cells, after prolonged treatments, the remaining survivors would have a higher proportion of the mutant. When this residual population is allowed to regrow, the mutant will comprise a higher proportion of the cells than in the previous cycle.

## Tolerance and Resistance Mutations Identified From Laboratory Evolution Experiments

We summarize the tolerance and resistance mutations identified from recent *in vitro* laboratory evolution experiments in Table 1. The mechanism of tolerance in the evolved populations appears to be dependent on the treatment conditions, including the bacterial growth phase at which the antibiotic is applied, the type of antibiotic used, and the durations of treatments. For example, overnight cultures of *E. coli* populations repeatedly diluted in a medium containing ampicillin eventually developed high tolerance by increasing their lag time (Fridman et al., 2014). Interestingly, the populations could match the lag time to the duration of antibiotic exposure, or in other words, optimizing their lag time depending on how they have been treated previously through unique mutations. A total of eight mutations were detected across all of their evolved strains. Reversion of three of these mutations restored the lag time of the ancestral strain. The mutated genes are in *vapB*, coding for an antitoxin of the *vapBC* TA module, *metG*, expressing methionyl-tRNA synthetase, and *prsA*, expressing ribose-phosphate diphosphokinase. The first two genes are involved in cellular components (TA modules and aminoacyl-transfer RNA synthetases, respectively) previously implicated in increased persistence (Gerdes and Maisonneuve, 2012; Germain et al., 2013; Kaspy et al., 2013). Although how exactly these mutations led to the extension in lag time is still unknown, quantitative analysis revealed that TA modules might act in a network manner to set the timescale of the lag-time distribution through regulation of the frequency and duration of growth arrest (Rotem et al., 2010). A follow-up study by the same group also revealed that prolonging the treatment cycles eventually caused the tolerant strains to attain resistance through mutations in the promoter of *ampC*, coding for a beta-lactamase associated with ampicillin resistance (Levin-Reisman et al., 2017). Another group that performed laboratory evolution experiments by treating stationary-phase *E. coli* with an aminoglycoside identified a different set of tolerance mutations, located in the genes *oppB, nuoN*, and *gadC*, expressing oligopeptide transport system permease protein, NADH-quinone oxidoreductase subunit N, and glutamate/gamma-aminobutyrate transporter, respectively (Van den Bergh et al., 2016). Their evolved strains showed no extension in lag time, and the tolerance phenotype could not be traced to a reduction in membrane potential (limiting antibiotic uptake) or translation activity (limiting target activity). Competition experiments in the presence of the antibiotic showed that the tolerant strains had a 160 to 360-folds fitness advantage compared to the ancestral strain over a single round of antibiotic treatment, which was primarily caused by a ∼1,000-folds increase in the rate of persister formation during the early stationary phase. Competition experiments in the absence of the antibiotic, on the other hand, showed that the mutants had reduced fitness compared to the ancestor, and the cost was caused by growth deficits linked to the increased proportion of persister cells. When regrown daily in the absence of antibiotic, the tolerant strains eventually reduced their tolerance to a level similar to the wild-type, due to additional compensatory mutations that increase fitness, rather than genetic reversion of the tolerance mutations.

**TABLE 1:** List of mutations identified from recent *in vitro* laboratory evolution experiments.

Study	Name of strain	Gene having mutations*	Phenotype	Phase of growth during treatment	Treatment duration (hours)	Antibiotic used during evolution
*Escherichia coli*						
Fridman et al., 2014	Tbl3a	*vapB*	Tolerance	Diluted Stationary phase	3	Ampicillin
	Tbl3b	*prsA*			3	
	Tbl5a	*metG, sspA*			5	
	Tbl5b	*vapB, pgm, yeaI*			5	
	Tbl8a	*prsA*			8	
Levin-Reisman et al., 2017	MGYE7-TOL	*prsA*	Tolerance	Diluted Stationary phase	4.5	Ampicillin
	MGYE7-TOLRES	*prsA, ampC*	Tolerance + Resistance			
	KLYE1-TOL	*metG*	Tolerance			
	KLYE1-TOLRES	*metG, ampC*	Tolerance + Resistance			
	EPECE7-TOL	*metG*	Tolerance			
	EPECE7-TOLRES	*metG, ampC*	Tolerance + Resistance			
Van den Bergh et al., 2016	Clone 1-1	*oppB*	Tolerance	Stationary phase	5	Amikacin
	Clone 2-1	*nuoN*				
	Clone 6-1	*gadC*				
Sulaiman and Lam, 2020a,b	Evo3A	*ybbA, yhgE, cyaA*	Tolerance	Exponential phase	3	Ampicillin
Sulaiman and Lam, 2020a	Evo3C	*mdoH, icd*				Ciprofloxacin
	Evo3P	*narZ, fusA*				Apramycin
Khare and Tavazoie, 2020	AC1	*leuS, murP*	Tolerance	Exponential phase	4	Ampicillin + Ciprofloxacin
	AC2	*selU, metG*				
	AC3	*yhjJ*				
	AC4	*ykgJ/ecpE, ribE, pth, yecD, cyaA*				
	AC5	*yecD, metG*				
	AC6	*ileS, ykgJ/ecpE, yecD*				
	AC7	*ykgJ/ecpE, ynfE, yecD, metG*				
	AC8	*proS, ykgJ/ecpE, yecD, yedR/yedS, yfcI*				
	AK1	*ompC, gltP*				Ampicillin + Kanamycin
	AK2	*pth*				
	AK3	*clpX/lon, pth*				
*Staphylococcus aureus*						
Mechler et al., 2015	D6	*pitA, gltS*	Tolerance	Stationary phase	7.5	Daptomycin

*The mutations shown include single point mutations, insertions and deletions. The exact location of the mutation may not be the same in different strains, although the gene where the mutation occurs is the same. For example, Tbl3b and Tbl8a both have single point mutations in the *prsA* gene, but the amino acid substitutions are R79H and C60Y in Tbl3b and Tbl8a, respectively.

In another study where exponential *E. coli* cells were repetitively treated with different classes of antibiotics (ampicillin, ciprofloxacin, and apramycin), the single point mutations detected from the three evolved populations were completely different, and their proteome profiles were also markedly divergent (Sulaiman and Lam, 2020a). Among the mutated genes, several have been previously linked to persistence and tolerance. For instance, the population repetitively treated with ampicillin bore a mutation in the *cyaA* gene, coding for adenylate cyclase. This enzyme is responsible for the formation of cyclic AMP (cAMP), which regulates genes involved in carbon catabolism, virulence, biofilm formation, and SOS response. It has also been reported that Δ*cyaA* mutants possess increased tolerance to β-lactams through the activation of oxidative stress responses and SOS-dependent DNA repair (Molina-Quiroz et al., 2018). On the other hand, the population treated with ciprofloxacin gained a mutation in *mdoH*, coding for glucosyltransferase H, an enzyme involved in the biosynthesis of periplasmic glucans. This protein was known to be important for biofilm-associated resistance of *Pseudomonas aeruginosa*, where the periplasmic glucans interact physically with antibiotics and prevent them from reaching their sites of action by sequestering the antibiotics in the periplasm (Mah et al., 2003). Lastly, the population from apramycin treatments had a mutation in *fusA*, coding for elongation factor G that catalyzes the ribosomal translocation step during translation elongation. It is also involved in ribosome assembly and recycling, and acts as a catalyst for the interconversion of (p)ppGpp, which plays a role in bacterial persistence. By cross-comparing the regulated proteomes of the three evolved populations generated from different antibiotic treatments, they identified protein candidates with similar expression profiles that might be important for tolerance. These include GrcA, a glycyl radical cofactor that have increased expression following the induction of toxin MazF; RaiA and RRF (ribosome recycling factor) which are related to ribosomal activity; AhpF, which protects the cell against DNA damage by alkyl hydroperoxides; NuoF, which plays a role in the electron transport chain; and CysP, a part of the ABC transporter complex. In a newer study where two antibiotics with orthogonal modes of action were used to repetitively treat exponential-phase *E. coli*, the time taken for the cells to develop tolerance is much longer compared to those trained with a single drug. The mutations observed in the tolerant populations were enriched in translation-related genes (*ileS, leuS, metG, proS*, and *pth*) (Khare and Tavazoie, 2020). Through transcriptional profiling, the authors identified overlapping pathways that were differentially regulated in the evolved strains compared to the wild-type. Gene ontology analysis revealed increased cellular stress in these persister-enriched populations, characterized by the up-regulation of the SOS response and phage shock genes, and the down-regulation of genes involved in core processes such as ATP production, electron transport chain, translation, cell division, and protein transport.

The tolerance mutations detected in laboratory evolution experiments across different laboratories are different and seemingly unrelated, perhaps due to the slight variations in the experimental conditions, the different ancestral strains used for the evolution experiments (with slight mutational variations), and/or the random nature of mutagenesis (Sulaiman and Lam, 2019). The mutated genes were involved in many and varied cellular functions. Evidently, tolerance can be enhanced by a large number of genetic changes throughout the genome, implying that many evolutionary pathways exist for the development of tolerance (Balaban and Liu, 2019). This is in contrast to resistance, which tends to arise due to the alterations in a few well-defined genes directly related to the action mechanism of the antibiotic. Moreover, the diversity of tolerance-associated genes discovered thus far suggested that tolerance may be better thought of the result of a perturbed biological network, and cannot be easily understood from a reductionist point-of-view, again in contrast to resistance. It is therefore not surprising that recent discoveries in the aforementioned laboratory evolution experiments have not led to a well-defined general mechanism for tolerance. Nonetheless, the two recent studies employing system-wide gene expression profiling by transcriptomics (Khare and Tavazoie, 2020) and proteomics (Sulaiman and Lam, 2020a) and cross-comparison of multiple tolerant mutants offer some hope that there might exist some common pathway(s) that underlie tolerance of various types, although much remains to be explored and clarified.

## Antibiotic Tolerance Boosts the Evolution of Resistance

There has been a debate whether antibiotic tolerance and resistance are two distinct phenomena or whether they have any connection at all. Recently, it has become clear that tolerance often precedes resistance in the course of evolution because tolerance mutations occur more frequently than resistance mutations, owing to the larger target size. When the treatment cycles of the evolution experiment were prolonged, Levin-Reisman and colleagues observed that tolerant *E. coli* populations eventually became resistant to the drug (Levin-Reisman et al., 2017). More alarmingly, they also showed that the tolerance mutations facilitated the development of resistance in the populations, and a positive epistatic interaction occurs between the tolerance and resistance mutations (Levin-Reisman et al., 2019). As the authors explained, the probability for the establishment of a mutation in cyclic antibiotic treatment depends on two main factors: the probability of mutation occurrence, and the probability that the mutation is not lost during antibiotic exposure. Tolerance increases the probability of the establishment of resistance mutations in two ways. It supports the continued survival of the population and hence extends the window of opportunity for rarer mutations to occur. At the same time, tolerance also increases the number of survivors, and therefore lowers the probability of resistance mutations getting lost during antibiotic exposure.

Other studies also corroborate the idea that antibiotic tolerance increases the chances for resistance mutations to develop. It was observed that the resistant mutants of *Mycobacterium tuberculosis* came from persisters (Sebastian et al., 2017), which are antibiotic tolerant. Persister cells in natural and laboratory *E. coli* strains have increased mutation rates which should promote the development of resistance (Windels et al., 2019a). Besides, a positive correlation between the number of persisters and the rate of resistance mutations was also noticed in *P. aeruginosa* (Vogwill et al., 2016). These observations of tolerance serving as a driver for resistance development seem to be general in a wide range of experimental setups and bacterial strains. Moreover, the high number of survivors in the tolerant populations, combined with the higher mutation rates due to the increased stress response in these tolerant cells, may act synergistically to increase the likelihood of the occurrence of resistance mutations (Windels et al., 2019b). Apart from genetic mutations in the bacterial chromosome (vertical transmission), resistance can also be acquired through the horizontal gene transfer of genetic elements. The high number of survivors of the tolerant mutant may serve as a reservoir to store the plasmids that would later be passed on to other cells when the antibiotic concentration has dropped. This was shown in a study where *Salmonella* persisters facilitated the spread of antibiotic resistance plasmids (Bakkeren et al., 2019). To make things worse, *in vivo*, these tolerant populations often remain in the host tissues in a dormant, non-growing state (such as in the case of *M. tuberculosis*), and may form biofilms that help them survive the antibiotic attack (Lewis, 2008).

## Clinical Detection of Tolerant Strains to Prevent the Development of Resistance

**FIGURE 1:**
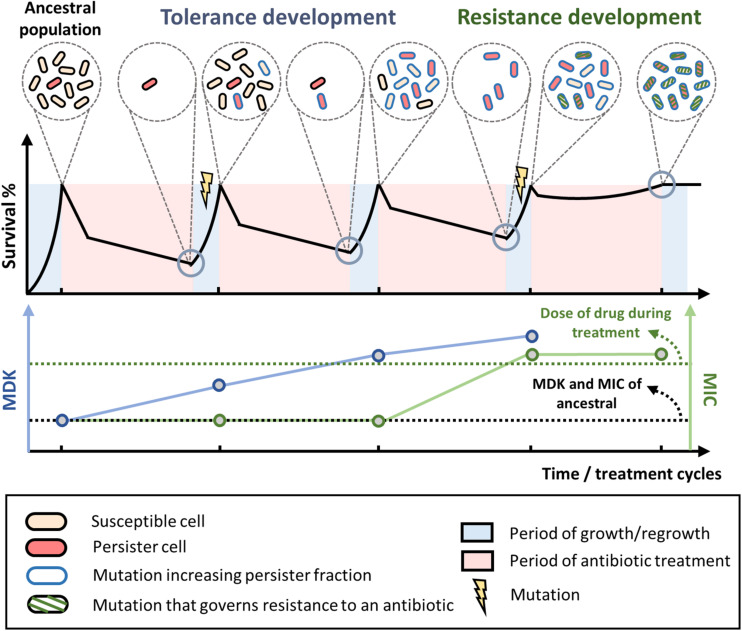
Tolerance and resistance development during antibiotic treatment and regrowth. Typical evolution of bacterial populations under repetitive antibiotic treatments. When treated with high doses of bactericidal antibiotics, biphasic killing is observed where the susceptible cells die rapidly while the persisters survive prolonged treatments. After a few treatment and regrowth cycles, mutations that increase tolerance or persistence fraction may occur (blue outline on the cells), leading to higher overall survival of the population and a higher minimum duration of killing (blue line) toward the antibiotic. Over more treatment cycles, the bacteria may eventually attain mutations that govern resistance to the antibiotic (green diagonal stripes on the cells). Once the MIC of the population (green line) exceeds the dose of the antibiotic used during the treatment (green dotted line), the population can grow within the treatment regime and the antibiotic is no longer effective. MIC, minimum inhibitory concentration. MDK, minimum duration of killing.

The typical process of tolerance and resistance development during laboratory evolution experiment is depicted in Figure 1. To a certain extent, this resembles the antibiotic therapy commonly adopted in clinics. While the tolerance level of the population (marked by an increase in MDK) keeps increasing over the treatment cycles, the MIC of the population stays the same. However, after the population has attained mutations that confer tolerance, it will greatly speed up the development of resistance and eventually cause the drug to be ineffective. Undetected tolerance is a bane to clinical practice, not only because the surviving cells can regrow and cause the relapse of diseases, but also because it facilitates the development of resistance. Unfortunately, the current standard in clinical practice is focused on screening for resistance through MIC testing. However, tolerance, which is not associated with an increased MIC, is overlooked. Based on the recent findings described here, it is evident that tolerance should also be screened to reduce the rate of resistance development. For patients who are receiving antibiotic therapy, the tolerance level of the pathogen should be monitored throughout the treatment period. Changing the drug or the treatment condition might be necessary once tolerance has been detected. Since tolerance mutations can be specific to the antibiotic used to treat the cells, switching the drug in time could have a positive impact on clinical outcomes. For example, it has been shown that the tolerance mutations in an evolved *E. coli* population from repetitive ampicillin treatment caused the cells to filament extensively during the treatment, thereby evading ampicillin targets (Sulaiman and Lam, 2020b). The single point mutations on the evolved population led to a perturbed biological network, which then activated the SOS response and suppressed the ROS generation in the cells, triggering filamentation. When treated with other antibiotics, the cells did not filament and the population died off. This highlights the importance for tolerance detection, as the tolerant population may readily be killed with another antibiotic, whereas continued treatment with the same antibiotic is not only ineffective but also dangerous because it promotes the development of resistance.

In another recent study of a patient with *methicillin-resistant S. aureus* (MRSA) bacteremia who received suppressive drug combination treatment, it was shown that resistance development is promoted when the bacteria has attained drug tolerance (Liu et al., 2020). The drug combination (daptomycin and rifampin) was shown to be effective in suppressing resistance development in the MRSA isolate when the cells were still sensitive to the drug. However, once the cells gained tolerance to daptomycin, the drug combination actually increased the chance for rifampin resistance to emerge. In other words, some drug combinations may be effective in preventing resistance development, but it needs to be applied before the cells develop tolerance. This again points to the importance of diagnostic tools for bacterial tolerance, which will help clinicians to devise suitable therapy that can prevent resistance development. Ideally, differential treatment for susceptible population, population harboring tolerance mutations, and population harboring resistance mutations should become standard practice, as they have distinct survival mechanisms against antibiotic assault.

Fast and easy tolerance detection methods such as TDtest (Gefen et al., 2017) and measurement of MDK_99_ (minimum duration for killing 99% of bacterial cells in the population) (Brauner et al., 2016) can be adopted in clinics. TDtest is a modification of the currently adopted disc diffusion assay for resistance detection. It comprises two steps. The first step is the standard resistance test where the antibiotic disk is applied on the agar plate to determine the inhibition zone. If this were the only step, tolerant cells that survive the transient antibiotic exposure would not be detected due to the lack of nutrients to support visible growth. Therefore, in the second step, the antibiotic disk is replaced by a nutrient disk to compensate for nutrient depletion, thus allowing the detection of tolerant cells, which otherwise would be regarded as susceptible in the standard disc diffusion assay. Another tolerance detection method similar to TDtest is the replica plating tolerance isolation system (REPTIS) (Matsuo et al., 2019). Instead of adding a nutrient disk, colony-forming units (CFUs) on the agar plate containing the antibiotic disk are transferred onto another plate without the antibiotic to allow bacterial growth. Regrowth of bacteria in the zone of inhibition shows the presence of tolerant cells. For rapid detection of tolerance that were caused by an increase in lag time (tolerance by lag), such as those observed in the study of Fridman et al. (2014), automated imaging with ScanLag (Levin-Reisman et al., 2010) or ColTapp (Bär et al., 2020) can be adopted.

## Future Directions in Studying Evolution of Bacterial Tolerance

Although the evolution of bacterial tolerance through repetitive antibiotic treatments has only been recently explored, we already know that tolerance and resistance can be developed in a much shorter timeframe than we previously thought. After merely 3 to 4 treatment cycles, the tolerance level of the treated population is already much greater compared to the ancestral population (Fridman et al., 2014; Sulaiman and Lam, 2020a). This rapid evolution warrants immediate attention from scientists. There is an urgent need to understand how bacteria could adapt so quickly to diverse treatment conditions, and how minor genetic alterations, in seemingly unrelated genes, can provide them with the means to survive antibiotic treatment. More extensive real-time studies of the evolution process of different bacteria toward different treatment conditions are needed, ideally using “omics” methodology that observes the cellular state at the systems level. For instance, a large-scale and high-throughput laboratory evolution study of different bacterial species toward different types of antibiotics should be conducted to comprehensively map the so-called “tolerome” (Brauner et al., 2016; Levin-Reisman et al., 2017), the collection of genes (and proteins) in which mutations affect the tolerance level of the cells. This will give us more insights into the bacteria’s adaptation mechanisms, and quicken the development of diagnostic tools. Going forward, more efforts should also be devoted to *in vivo* studies of this phenomenon, since findings in *in vitro* experiments may not directly translate to the host environment which is more complex and heterogeneous. Although laboratory experiments and theoretical predictions showed that bacterial populations could gain high levels of tolerance after a few cycles of repetitive antibiotic treatments (survival ranging from 10 to 100%), pathogenic isolates from patients after frequent antibiotic treatments often do not reach the predicted tolerance levels. It may be because the present models fail to capture some of the “hidden” costs associated with tolerance in the hostile environment of the host (Van den Bergh et al., 2017). Additional factors such as host defense and species competition may also come into play *in vivo* (Sakoulas et al., 2017; Sulaiman and Lam, 2019). Although models for *in vivo* evolution are still lacking, researchers have been performing longitudinal studies of bacteria strains isolated from patients, thereby revealing the dynamics of tolerance evolution within the host (Liu et al., 2020). Such studies are highly valuable. In addition, for patients with severe and recalcitrant infections, combinatorial treatment is often employed (Liu et al., 2020), while most of the reported *in vitro* evolution experiments were limited to a single drug. A laboratory evolution experiment that used drug combination to treat *E. coli* populations showed that longer treatment cycles are required for the populations to finally achieve tolerance, suggesting different evolutionary dynamics (Khare and Tavazoie, 2020). Future laboratory evolution experiments should take into account the use of drug combinations to better simulate clinical conditions, which in theory should be more complicated as drug combinations could act in a suppressive or synergistic manner.

## Author Contributions

Both authors listed have made a substantial, direct and intellectual contribution to the work, and approved it for publication.

## Conflict of Interest

The authors declare that the research was conducted in the absence of any commercial or financial relationships that could be construed as a potential conflict of interest.

## Supplementary Material

Supplementary Figure 1 Evolutionary model simulates the invasion of small number of tolerant mutants after repetitive antibiotic treatment. **(a)** Mathematical equations of the evolutionary model, which is an extension of the model of persistence. Unlike the model of persistence that assumes two subpopulations, there are four subpopulations in the evolutionary model; wild-type normal population (*n*_*W*_ (*t*)), wild-type persisters population (*p*_*W*_ (*t*)), mutant normal population (*n*_*M*_ (*t*)), and mutant persisters population (*p*_*M*_ (*t*)). **(b)** The evolutionary model was used to simulate typical laboratory evolution experiments. After a few cycles of antibiotic treatment, the population will have increased survival to the antibiotic. The shaded regions are the period of high-dose antibiotic treatment. The right figure shows that the number of persisters is very low in the ancestral population, which is susceptible to the antibiotic. However, after a few cycles, the small number of tolerant mutants gradually take over the population, which in the end becomes tolerant.
