# Supplementary figures and images for: Evolution of Bacterial Tolerance Under Antibiotic Treatment and Its Implications on the Development of Resistance
Source: Front Microbiol. 2021 Feb 26;12:617412. doi: 10.3389/fmicb.2021.617412 (PMC7952611; doi:10.3389/fmicb.2021.617412)

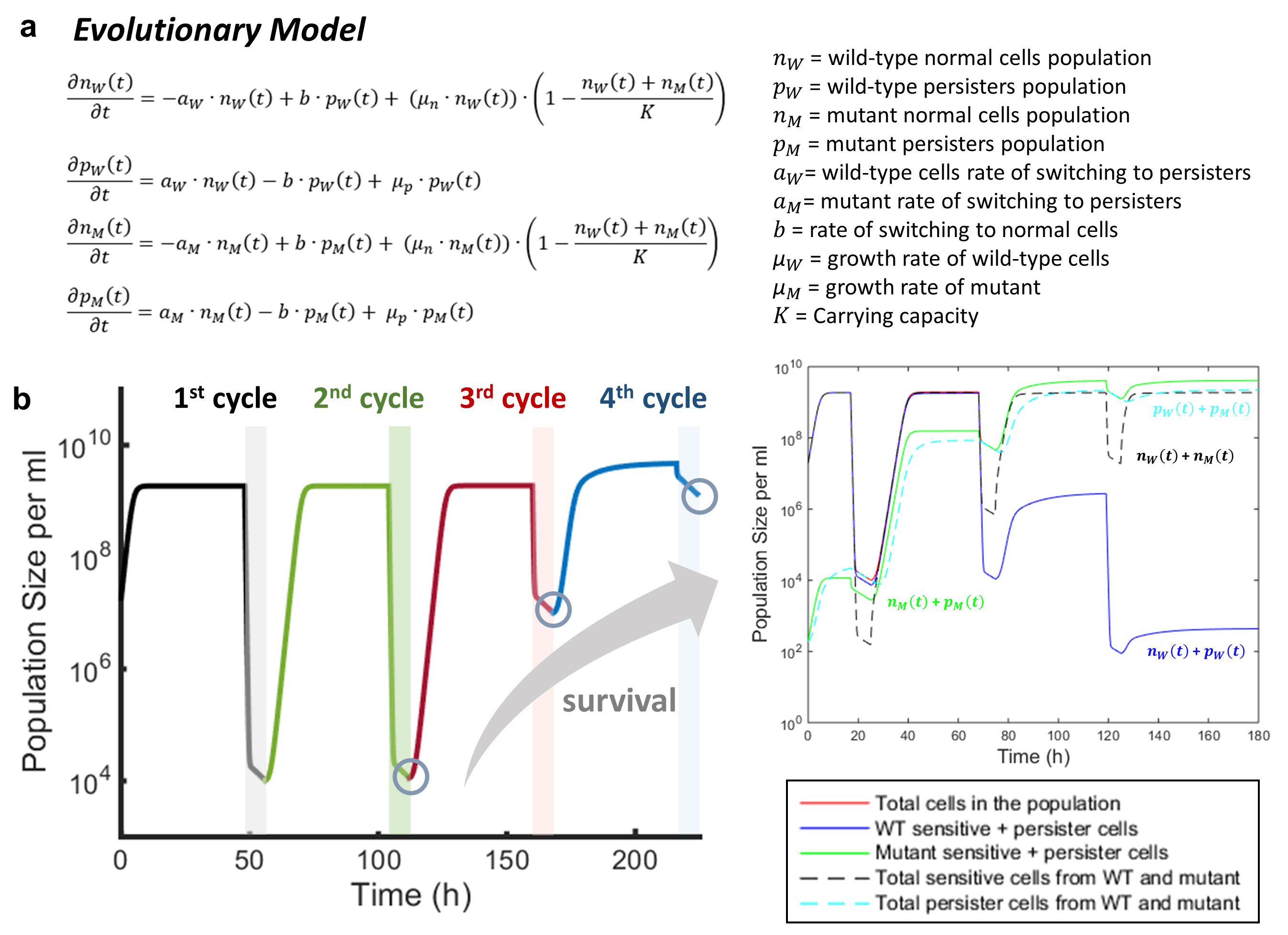

Supplement: Supplementary Figure 1 — Evolutionary model simulates the invasion of small number of tolerant mutants after repetitive antibiotic treatment. (a) Mathematical equations of the evolutionary model, which is an extension of the model of persistence. Unlike the model of persistence that assumes two subpopulations, there are four subpopulations in the evolutionary model; wild-type normal population (nW (t)), wild-type persisters population (pW (t)), mutant normal population (nM (t)), and mutant persisters population (pM (t)). (b) The evolutionary model was used to simulate typical laboratory evolution experiments. After a few cycles of antibiotic treatment, the population will have increased survival to the antibiotic. The shaded regions are the period of high-dose antibiotic treatment. The right figure shows that the number of persisters is very low in the ancestral population, which is susceptible to the antibiotic. However, after a few cycles, the small number of tolerant mutants gradually take over the population, which in the end becomes tolerant. [file Image_1.TIF]
